# Interplay of Mre11 Nuclease with Dna2 plus Sgs1 in Rad51-Dependent Recombinational Repair

**DOI:** 10.1371/journal.pone.0004267

**Published:** 2009-01-23

**Authors:** Martin E. Budd, Judith L. Campbell

**Affiliations:** Divisions of Biology and Chemistry, Caltech, Braun Laboratories, Pasadena, California, United States of America; National Cancer Institute, United States of America

## Abstract

The Mre11/Rad50/Xrs2 complex initiates IR repair by binding to the end of a double-strand break, resulting in 5′ to 3′ exonuclease degradation creating a single-stranded 3′ overhang competent for strand invasion into the unbroken chromosome. The nuclease(s) involved are not well understood. Mre11 encodes a nuclease, but it has 3′ to 5′, rather than 5′ to 3′ activity. Furthermore, mutations that inactivate only the nuclease activity of Mre11 but not its other repair functions, *mre11-D56N* and *mre11-H125N*, are resistant to IR. This suggests that another nuclease can catalyze 5′ to 3′ degradation. One candidate nuclease that has not been tested to date because it is encoded by an essential gene is the Dna2 helicase/nuclease. We recently reported the ability to suppress the lethality of a *dna2Δ* with a *pif1Δ*. The *dna2Δ pif1Δ* mutant is IR-resistant. We have determined that *dna2Δ pif1Δ mre11-D56N* and *dna2Δ pif1Δ mre11-H125N* strains are equally as sensitive to IR as *mre11Δ* strains, suggesting that in the absence of Dna2, Mre11 nuclease carries out repair. The *dna2Δ pif1Δ mre11-D56N* triple mutant is complemented by plasmids expressing Mre11, Dna2 or dna2K1080E, a mutant with defective helicase and functional nuclease, demonstrating that the nuclease of Dna2 compensates for the absence of Mre11 nuclease in IR repair, presumably in 5′ to 3′ degradation at DSB ends. We further show that *sgs1Δ mre11-H125N*, but not *sgs1Δ*, is very sensitive to IR, implicating the Sgs1 helicase in the Dna2-mediated pathway.

## Introduction

Double-strand breaks (DSBs) are a major result of endogenous DNA replication errors and of exogenous DNA damaging agents, and failure to repair such breaks leads to the gross chromosomal rearrangements characteristic of tumor cells. In yeast, homologous recombination (HR), carried out by proteins encoded by the *RAD52* epistasis group, is used to repair DSBs induced by ionizing radiation (IR). The time course of IR repair in vivo has been assayed by observing the post-irradiation appearance of foci of the various HR repair proteins tagged with green fluorescent protein [1]. Mre11, Rad50, and Xrs2 form a complex (MRX) and are the first arrivals after DSB formation. The MRX complex regulates the processing of the end of the DSB, resulting in resection of the 5′ end and leaving a 3′ single-stranded overhang [2]. RPA binds the single-stranded DNA, followed by Rad52 and Ddc2/Mec1 binding. Ddc2/Mec1 is the apical checkpoint-activating protein kinase. Rad52 can mediate the exchange of RPA for the Rad51 strand exchange protein, generating the 3′ terminal filament that initiates invasion into the intact sister or homologous chromosome [3]. Rad51 association is followed by the binding of Rad54 and additional strand exchange proteins, Rad55 and Rad57.

Mechanistically, the initial steps are poorly understood. Our studies address the function of the MRX complex, which has ATP binding, ATPase, adenylate kinase, nuclease, and DNA end-bridging activity [4], [5]. Mre11 houses the nuclease activity in a domain containing five phosphodiesterase motifs homologous to the *E. coli* sbcD exonuclease. Mre11 has 3′ to 5′ exonuclease activity on double-stranded DNA and single-strand endonuclease activity on DNA hairpins [6]–[8]. Rad50 is an ATP binding protein, with the Walker A motif in the N terminus and Walker B motif in the C terminus separated by a long heptad repeat domain [9]. A signature (arginine finger) region in the C terminus is also part of the ATP binding motif [10]. Mutations in the ATP binding motif result in strains with meiotic recombination null phenotypes [9], [11]. The Xrs2 protein (known as Nbs1 in mammals) contains a forkhead domain at the N terminus and a binding domain for Tel1 at the C-terminus. Tel1 is a kinase involved in telomere maintenance and checkpoint signaling. Mre11/Rad50/Nbs1 has weak ATP-stimulated DNA unwinding activity [12].

In bacterial recombinational repair, the strand invasion intermediate is generated by the RecBCD helicase/nuclease in a complicated reaction requiring 5′ to 3′ helicase, 3′ to 5′ helicase, 5′ to 3′ nuclease, 3′ to 5′ nuclease, and a specific DNA sequence called chi. A counterpart to this complex has not been identified in eukaryotic cells. The most highly radioresistant organism known, *Micrococcus radiodurans*, does not have a RecBCD helicase/nuclease, but does possess a complete RecF pathway [13]. A major question has been what pathway is found in eukaryotes. *MRE11* is a nuclease involved in DSB repair and *mre11Δ* strains are extremely sensitive to irradiation. After 17 krads the survival of an *mre11Δ* strain is about 0.06%. If one assumes 2 krads results in 1 DSB per haploid cell [14], then a survival of about 0.02% would be expected in the absence of repair. Therefore, repair is nearly absent in a *mre11Δ* strain. *mre11-D65N* and *mre11-H125N* are more sensitive to IR than *MRE11* strains but much more resistant than *mre11Δ* strains. At a high dose of 70 krads about 75 DSBs are induced, which would predict a survival value of e^−35^ or 10^−13^% in the complete absence of repair. The survival of *mre11-D56N* and *mre11-H125N* mutants at 70 krads is about 5 to 10 fold less than *MRE11* strains (3% survival), which is much higher than the predicted survival in the absence of DSB repair [15], [16]. In addition, paradoxically, Mre11 has 3′ to 5′ exonuclease activity rather than the 5′ to 3′ nuclease expected for 5′ resection [6]–[8]. Furthermore, resection is not reduced in *mre11* mutants defective in the nuclease active site but proficient in the other functions of Mre11 [17], [18]. This has led to the proposal that other nucleases are required and that they can compensate for the absence of the Mre11 nuclease. Sae2 is another nuclease that appears to be associated with the MRX complex, but also has 3′ to 5′ activity [19]. Exo1, a 5′ to 3′ nuclease of the Fen1 family involved in mismatch repair, may play a role. Overproduction of Exo1 indeed increases the IR resistance of an *mre11Δ* strain [20], [21]. However, *exo1Δ mre11Δ* and *exoΔ mre11-nuclease deficient* double mutants are viable, are no more sensitive to IR than *mre11Δ* or *mre11-nuclease deficient* mutants, respectively, and can carry out gene conversion as efficiently in vivo as *EXO1 MRE11* strains [20]. This suggests that yet another nuclease must compensate for Mre11 nuclease deficiency.

Dna2 is a candidate for this role. Dna2 is a 5′ to 3′ helicase, 5′ to 3′ exo/endonuclease, 3′ to 5′ exo/endonuclease, and has single strand DNA annealing and strand exchange activity [22]–[28]. Dna2 plays a role in Okazaki fragment processing (OFP), assisting FEN1 in RNA primer removal [26], [29]. *dna2* mutants, however, are extremely sensitive to bleomycin and IR, consistent with an additional role in DSB repair [30]–[32]. Also, we showed many years ago that *dna2-2 rad50-5* mutants are synergistically defective in IR repair [31]. Finally, Dna2 forms a hub in a genetic network of 322 proteins that preserve genome stability [33]. Since the *DNA2* gene is essential, however, it has been difficult to test the hypothesis that Dna2 nuclease participates in generation of the 3′ overhang during DSB repair. Recently, we found that the inviability of *dna2Δ* mutants is efficiently suppressed by deletion of Pif1, a helicase orthologous to *E. coli* RecD helicase, that functions in DNA replication and telomere homeostasis [34], [35]. Using a *dna2Δ pif1Δ* strain and *mre11* nuclease defective mutants, we show that the nucleases of Dna2 and Mre11 are required in repair of IR-induced damage, presumably in 5′ resection, and that they can compensate for each other's loss. In addition, we show that the helicase of Dna2 is dispensable but that the Sgs1 helicase is required for Dna2 nuclease to be able to compensate for lack of Mre11 nuclease.

## Results

### Dna2 nuclease can compensate for the loss of Mre11 nuclease in DSB repair and vice versa

**Figure 1:**
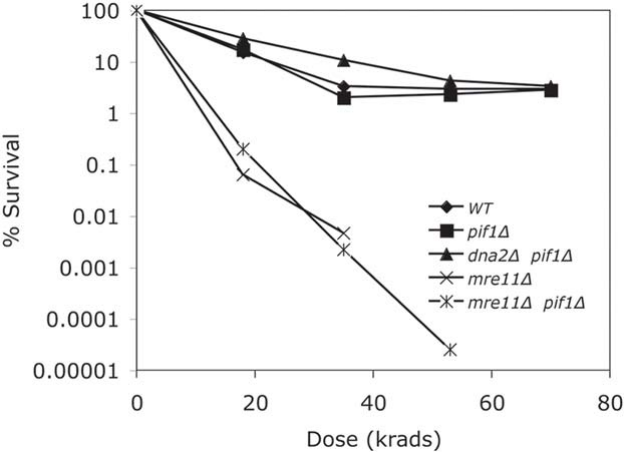
X-ray resistance of the *dna2Δ pif1Δ* strain compared to sensitivity of *mre11*Δ *pif1Δ*. The strains MB120-5A (WT), MB121- *pif1Δ*, MB161B- *dna2Δ pif1Δ*, MB122-17C- *mre11Δ*, MB124-2D- *mre11Δ pif1Δ* were harvested in mid-log phase, resuspended in water, irradiated, serially diluted (1∶10), and plated.

The relative importance of repair proteins can be measured by determining the survival of mutants defective in the respective proteins after increasing doses of a DNA damaging agent. We have used assays that measure survival after an acute dose of IR followed by growth in the absence of the DNA damaging agent. We were surprised that the *dna2Δ pif1Δ* mutant was resistant to X-rays (Figure 1, the sensitivity of *mre11Δ and mre11Δ pif1Δ* are shown as controls), since several *dna2* hypomorphic mutants are sensitive to IR [31], [36]. This suggested that in the absence of Pif1, another protein might carry out the role of Dna2 in repair. Both hypomorphic *dna2* mutants and the *dna2Δ pif1Δ* strain are synthetically lethal with mutants affecting three nuclease mutants defective in DSB repair, *mre11Δ*, *sae2Δ*, and *exo1Δ* [31], [33], [34]. We reasoned that the Mre11 nuclease might be compensating for the loss of Dna2 during X-ray repair in the *dna2Δ pif1Δ* strain.

**Figure 2:**
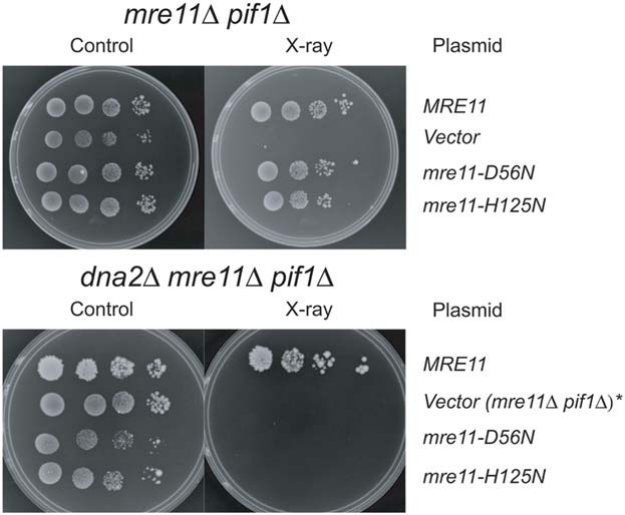
*dna2Δ pif1Δ mre11Δ* (pRS414*mre11-D56N*) and *dna2Δ pif1Δ mre11Δ* (pRS414*mre11-H125N*) strains exhibit similar sensitivity to X-rays as *mre11Δ pif1Δ*. These strains were created by dissecting tetrads from the following crosses: *MATa dna2Δ pif1Δ trp1Δ bar1Δ*/ *MATα mre11Δ trp1Δ* (pRS414*MRE11*), *MATa dna2Δ pif1Δ trp1Δ bar1Δ/ MATα mre11Δ trp1Δ* (pRS414*mre11-D56N*) and *MATa dna2Δ pif1Δ trp1Δ bar1Δ*/ *MATα mre11Δ trp1Δ* (pRS414*mre11-H125N*) diploids. Trp^+^ spores were replica plated, and irradiated. Control strains *mre11Δ pif1Δ* (pRS414*MRE11*) and *dna2Δ pif1Δ mre11Δ* (pRS414*MRE11*) were identified from the respective cross and were X-ray resistant. *dna2Δ pif1Δ mre11Δ* (pRS414*mre11D56N*) or *dna2Δ pif1Δ mre11Δ* (pRS414*mre11H125N*), from the two respective crosses were radiation sensitive. To compare the radiation sensitivity of the *dna2Δ pif1Δ mre11Δ* (pRS414*mre11-D56N*) and the *dna2Δ pif1Δ mre11Δ* (pRS414*mre11-H125N*) (bottom panel) to *mre11Δ pif1Δ* (pRS414*mre11-D56N*) and to *mre11Δ pif1Δ* (pRS414*mre11-H125N*) strains (top panel), serially diluted cells were irradiated with 26 krads on a plate and allowed to grow for three days. Note that a *dna2Δ pif1Δ mre11Δ* strain carrying only the pRS vector is inviable, so an *mre11Δ pif1Δ* strain carrying the pRS vector alone is shown as the control, as indicated by the * in the bottom panel.

To test the hypothesis that Mre11 and Dna2 had compensatory functions, we wished to compare the X-ray sensitivity of *dna2Δ pif1Δ* and *dna2Δ pif1Δ mre11-nuclease defective* strains. We first created *dna2Δ pif1Δ mre11Δ* strains carrying *MRE11*, *mre11-D56N* or *mre11-H125N* on centromeric plasmids. The D56N and H125N mutations lie in the conserved phosphodiesterase motif of Mre11, and the mutant proteins have no in vitro endonuclease activity [18], [37]; however, they are able to form a complex with Rad50 and Xrs2. As shown in Figure 2, both wild-type and nuclease-defective alleles of *MRE11* suppress the lethality of *dna2Δ pif1Δ mre11Δ* strains. As predicted, however, the strains deficient in both Dna2 and the Mre11 nuclease are X-ray sensitive, while strains expressing wild-type Mre11 are resistant to IR. Thus, in the absence of Dna2, Mre11 nuclease is required for X-ray repair. By contrast, the X-ray sensitivity of *pif1Δ mre11Δ*, in which there is a functional Dna2 nuclease, is complemented not only by wild-type *MRE11* but also by nuclease defective *mre11-D56N* or *mre11-H125N*. These results suggest that Dna2 nuclease can function in X-ray repair in the absence of Mre11 nuclease, but not in the complete absence of Mre11. It also says that Mre11 nuclease can function in X-ray repair in the absence of Dna2.

**Figure 3:**
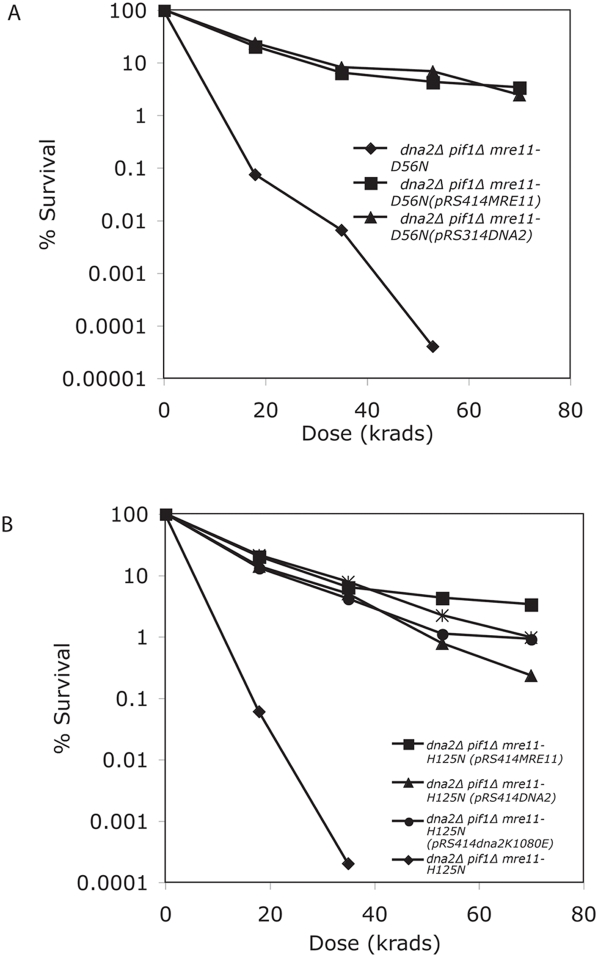
(A) X-ray sensitivity of a *dna2Δ pif1Δ mre11-D56N* strain and complementation by CEN plasmids containing Mre11 or Dna2. DNA fragments containing *mre11-D56N* and *mre11-H125N* mutant genes with 800 bp 5′ and 140 bp 3′ to the gene were cloned into pRS306*URA3*, cleaved in the *mre11* promoter and transformed into a *dna2*Δ *pif1*Δ *MRE11* strain to allow integration at the *MRE11* locus. The resulting transformants were propagated on YPD media then grown on 5-FOA containing media to excise the *MRE11* allele. Approximately 15% of the pRS306*mre11-D56N* and pRS306*mre11-H125N* Ura^−^ pop outs were X-ray sensitive. Strains MB161B-56- *dna2Δ pif1Δ mre11D56N*, MB161B-56 (pRS414*MRE11*), MB161B (pRS314*DNA*2) were then compared for X-ray sensitivity as in Figure 1. (B) X-ray sensitivity of a *dna2Δ pif1Δ mre11-H125N* strain and complementation by plasmids containing *MRE11* or nuclease proficient *DNA2* genes. Strain MB161B-125 – *dna2Δ pif1Δ mre11H125N*, MB161B-125 (pRS414*MRE11*), MB161B-125 (pR314*DNA2*), MB161B (pRS314*dna2K1080E*), MB161B-125 (pRS314*dna2-2*) were compared for X-ray sensitivity as described in Figure 1.

To insure that the X-ray sensitivities of the nuclease-dead *mre11* mutants were not the result of inefficient expression due to employing extrachromosomal plasmids to express the *mre11* alleles, the *mre11* nuclease defective genes were integrated into the chromosome by two-step gene replacement. The *dna2Δ pif1Δ mre11-D56N* and *dna2Δ pif1Δ mre11-H125N* clones were as X-ray sensitive as the *mre11Δ pif1Δ* strain (compare Figure 3A and Figure 3B to Figure 1). Importantly, in both *dna2Δ pif1Δ mre11-D56N* and *dna2Δ pif1Δ mre11-H125N* X-ray sensitive clones, X-ray resistance is restored by introduction of a plasmid expressing either *DNA2* or *MRE11* (Figure 3A,B). The helicase activity of Dna2 was not essential for complementation, since the X-ray resistance of the *dna2Δ pif1Δ mre11-H125N* clone was restored by plasmids expressing Dna2 with either of two mutations in the helicase domain: pRS314*dna2K1080E* plasmid, which has a mutation in the conserved Walker P loop but has a functional nuclease, and pRS314dna2R1253Q (*dna2-2*) (Figure 3B). We conclude that it is the nucleolytic activities of Mre11 and Dna2 that are complementary.

**Figure 4:**
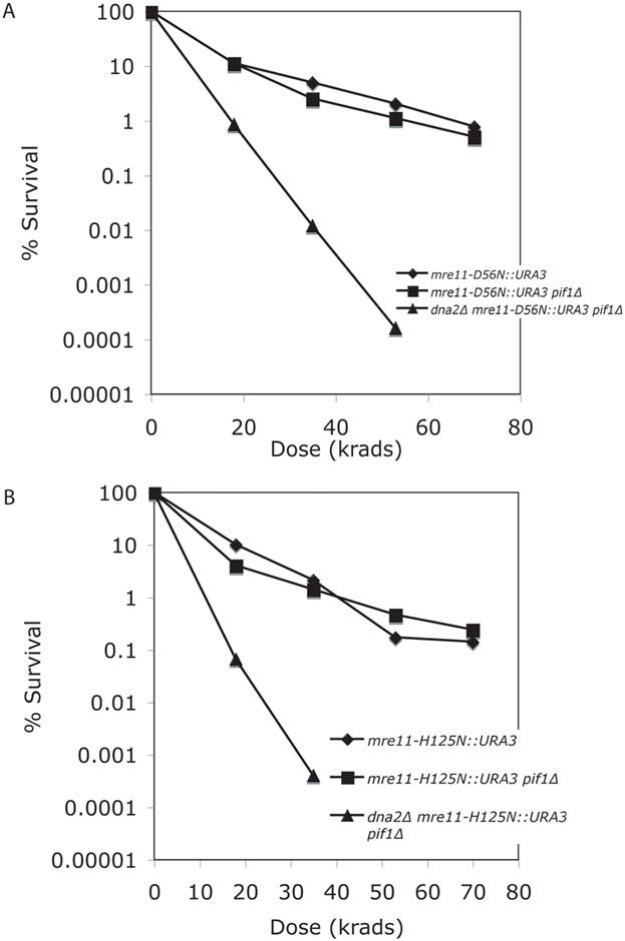
(A) Comparison of the X-ray sensitivity of a *mre11-D56N pif1Δ* strain with a *dna2Δ mre11-D56N pif1Δ* strain. The integrating plasmids pRS306*mre11-D56N::URA3* and *pRS306mre11-H125N::URA3* were cut with Sph1 which cleaves about 300 bp 5′ to the ATG of *MRE11* and transformed into a *MATa mre11Δ::natR* strain and insertion at the *MRE11* locus was selected for on G418 plates lacking uracil. These strains were mated with MB161B-*MATa dna2Δ pif1*Δ *MRE11*, and the diploids were sporulated and dissected. When the resulting spores were scored, the NatR gene always segregated with the *URA3* gene, demonstrating that the pRS306mre11nuclease minus plasmids were integrated at the *MRE11* loci in the respective strains. Strains MB126-*mre11Δ::natR::mre11D56N::URA3*, MB128- *mre11Δ::natR::mre11-D56N::URA3 pif1Δ*, and MB133 - *dna2Δ pif1Δ mre11Δ::natR::mre11-D56N::URA3* were treated as in Figure 1 to determine IR sensitivity. (B) Comparison of the X-ray sensitivity of a *mre11-H125N pif1Δ* strain with a *dna2Δ mre11-H125N pif1Δ* strain. Strains were constructed as in Figure 4A. Strains MB127-*mre11Δ::natR::mre11-H125N::URA3*, MB129-*mre11Δ::natR::mre11-H125N::URA3 pif1Δ*, and MB134 - *dna2Δ mre11Δ::natR::mre11-H125N::URA3 pif1Δ*, were treated as in Figure 1.

*mre11-D56N* and *mre11-H125N* mutants are not sensitive enough to DNA damaging agents to allow direct scoring in genetic crosses. Therefore, to compare the *DNA2 PIF1 mre11 nuclease-minus* with the *dna2Δ pif1Δ mre11 nuclease-minus* mutants, triple mutants were also constructed by introduction of a genetically tagged copy of the *mre11* nuclease mutant genes into the chromosome, as described in the legend to Figure 4. *dna2Δ pif1*Δ *MRE11* were X-ray resistant (see Figure 1). As before, *dna2Δ pif1Δ mre11Δ*::*NatR::306 mre11-D56N::URA3* or *dna2Δ pif1Δ mre11Δ*::*NatR::306 mre11-H125N*::*URA3* were highly sensitive to IR compared to the *mre11Δ::NatR::306 mre11D56N*::*URA3* and the *mre11Δ*::*NatR*::306 *mre11H125N*::URA3 strains (Figure 4 A,B). Clearly, in the absence of Dna2 nuclease, Mre11 nuclease is essential for repair of IR-induced damage.

### Sgs1 is Required for the Dna2-mediated Repair Pathway

**Figure 5:**
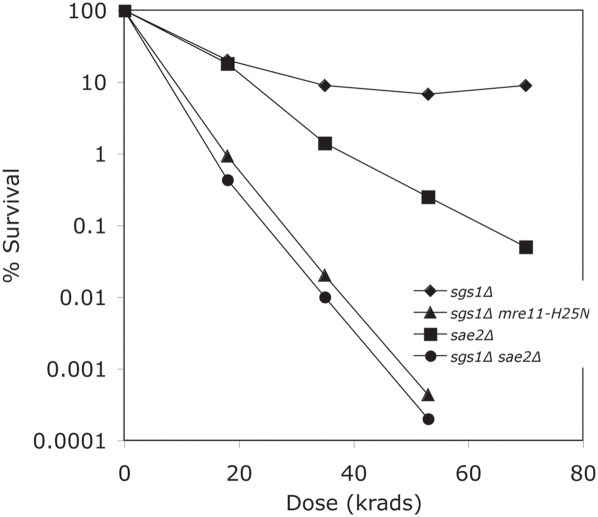
Comparison of the X-ray sensitivity of strain *sgs1Δ* with *sgs1Δ mre11-H125N* and *sgs1Δ sae2Δ*. Strains MB137 *sgs1Δ*, MB138 *sgs1Δ mre11-H125N* and MB135 *sae2Δ* and MB136 *sgs1Δ sae2Δ* were constructed in this study and treated as in Figure 1.

Based on the above results, we hypothesized that Dna2 nuclease might play a role in resection of the 5′ end at IR-induced breaks. Since the Dna2 helicase was not required, we asked what helicase might participate to convert the DSB into a substrate recognized by Dna2, which recognizes only single-stranded termini. Sgs1, which encodes a helicase of the RecQ family, was a likely candidate, since helicase defective *dna2-2* mutants are defective in X-ray repair but the double *sgs1Δ dna2-2* mutant is significantly more sensitive to IR than *dna2-2*, even though *sgs1Δ* itself is X-ray resistant [31], [38]. Furthermore, we also recently demonstrated that human BLM, a RecQ family helicase, can suppress the MMS and bleomycin sensitivity of *dna2-2* mutants [39], [40]. We therefore investigated whether yeast Sgs1 might be involved in the Dna2 repair pathway. We found, as have others, that *sgs1Δ* mutants are as resistant to IR as wildtype (Figure 5). We then constructed an *sgs1Δ mre11-H125N* double mutant. As we predicted, the double mutant was as sensitive to IR as the *dna2Δ mre11-H125N* mutant (compare Figures 3 and 4 with Figure 5). We conclude that Sgs1 is required in order for Dna2 to compensate for the loss of Mre11 nuclease during repair of IR-induced damage.

Sae2 is also a nuclease that functions in the MRX complex and can function as a nuclease on its own [19]. Thus, we wanted to test whether *sae2Δ sgs1Δ* strains have the same survival as *sgs1Δ mre11-H125N*, *dna2Δ mre11-H125N*, and *mre11Δ* strains. As illustrated in Figure 5, *sae2Δ sgs1Δ* strains are indeed as sensitive to IR as *sgs1Δ mre11-H125N* strains. These results put Mre11 nuclease and Sae2 nuclease in one pathway and Dna2 nuclease and Sgs1 helicase in another pathway in the initiation of exonucleolytic degradation after DSB formation.

### Dna2 Helicase Activity produces the Requirement for Dna2 Nuclease Activity

Separation of function of MRX ATP binding and nuclease functions has been investigated extensively so we attempted a similar analysis of Dna2. The nuclease of Dna2 maps to a region N-terminal to the helicase, between amino acids 649–744 in a domain containing conserved *E. coli* RecB nuclease motifs. Two mutants of Dna2, D657A and E675A, specifically affecting RecB active site motifs, result in Dna2 proteins with greatly reduced or undetectable nuclease activity but with intact helicase and ATPase activity [25], [41]. The plasmid, p*GAL::DNA2*, is a high copy vector containing a *GAL10* inducible Dna2 but expresses sufficient Dna2 without induction to complement *dna2-1* mutants at 37°C on glucose plates [25]. The plasmids p*GAL*::*dna2-K1080E*, p*GAL*::*dna2-D657A* p*GAL*::*dna2-E675A* do not complement a *dna2-1* mutant on glucose plates at 37°C [25]. Also, we found here, that the *dna2-K1080E*, *dna2-D657A*, and *dna2-E675A* genes expressed from centromeric plasmids fail to complement the *dna2Δ PIF1* mutant (data not shown). This indicates that the ATP binding and nuclease functions of Dna2 are essential.

**Figure 6:**
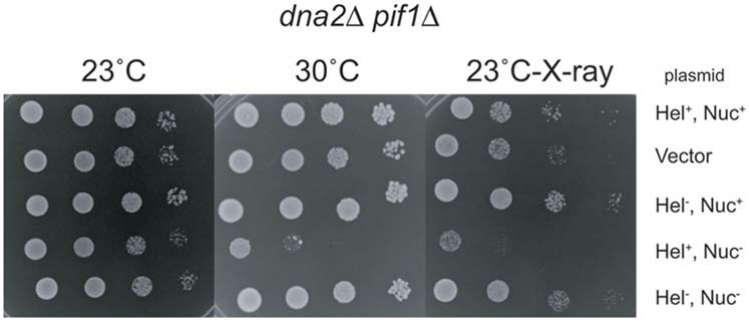
Expression of nuclease deficient Dna2 is toxic in a *dna2Δ pif1Δ* strain. Strain MB21-*dna2Δ pif1Δ* carrying either pRS314*DNA2*, Hel^+^, Nuc^+^; pRS314, Vector; pRS314*dna2K1080E*, Hel^−^Nuc^+^; pRS314*dna2E675A*, Hel^+^Nuc^−^ or pRS314*dna2K1080E*,*dna2E675A*, Hel^−^Nuc^−^ plasmids was grown, serially diluted 10 fold and plated on trptophan deficient plates with or without 26 krads IR, and allowed to grow for four days at the temperatures indicated.

To verify that an active Dna2 nuclease was necessary for IR repair in the absence of Mre11 nuclease, we compared the growth and X-ray sensitivity of *dna2Δ pif1Δ* strains transformed with centromeric plasmids carrying *DNA2*, *dna2K1080E*, and *dna2E675A*, or *dna2K1080E*,*E675A*. The *dna2K1080E*,*E675A* is doubly deficient in both helicase and nuclease. When the *dna2Δ pif1Δ* transformants were incubated at 23°C on tryptophan deficient plates all five transformants grew, as illustrated in Figure 6. At 30°C, the transformants containing the plasmids pRS314, pRS314*DNA2*, pRS314*dna2K1080E* (helicase minus, nuclease plus) formed viable colonies. Thus, *pif1Δ* suppresses the inviability of a *dna2Δ PIF1* (pRS314*dna2-K1080E*) strain, and in fact the *dna2Δ pif1Δ* (RS314*dna2-K1080E*) strain even grows at 37°C, unlike the *dna2Δ pif1Δ* strain [34]. Interestingly, the pRS314*dna2E675A* transformant (helicase plus, nuclease minus) did not form colonies at 30°C (Figure 6). However, the *pRS314dna2K1080E*,*E675A* (helicase minus, nuclease minus) did support viability at 30°C. We conclude that at this temperature the absence of the nuclease was deleterious to normal DNA replication, and furthermore that it is the Dna2 helicase activity that generates a need for the Dna2 nuclease.

Next, the *dna2Δ pif1Δ* transformants were irradiated after plating and incubated at 23°C. The pRS314, pRS314*DNA2*, pRS314*dna2K1080E*, and pRS314*dna2K1080E*,*E675A* transformants were resistant to IR, whereas the transformant containing the pRS314*dna2E675A* plasmid was sensitive to IR. Thus, as with growth at the restrictive temperature of 30°C, after IR, the Dna2 nuclease minus strain is sensitive to damage, but only in the presence of an active Dna2 helicase. We conclude that the activities of the Dna2 helicase and nuclease must be properly coordinated for both DNA replication and repair.

**Figure 7:**
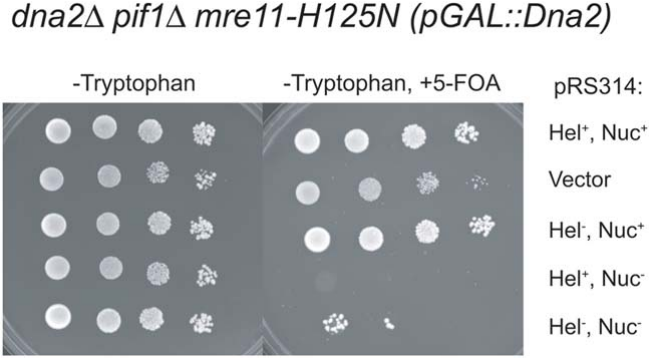
Inactivation of the Dna2 nuclease is lethal in a *pif1Δ mre11-H125N* strain. A *dna2Δ pif1Δ mre11-H125N trp1Δ* (p*GAL*::*DNA2URA3*) strain was transformed with the Trp^+^ plasmids pRS314*DNA2*, Hel^+^, Nuc^+^; pRS314, Vector; pRS314*dna2K1080E*, Hel^−^Nuc^+^; pRS314*dna2E675A*, Hel^+^Nuc^−^ or pRS314*dna2K1080E*,*dna2E675A*, Hel^−^Nuc^−^. The Trp^+^Ura^+^ transformants were grown in tryptophan deficient media and spotted onto tryptophan minus (left) or tryptophan minus, 5-FOA containing (right) media at 23°C and photographed after 4 days at 23°C.

The requirement for coordination of the Dna2 helicase and nuclease was further examined in the *dna2Δ pif1Δ mre11-H125N* mutant. The *dna2Δ pif1Δ mre11-H125N* (p*GAL*::*DNA2*) was transformed with the same set of Dna2 mutant plasmids used in Figure 6, to yield double transformants, *dna2Δ pif1Δ mre11-H125N* (p*GAL*::*DNA2*) (pRS414-*dna2* mutant). Each double transformant was grown in tryptophan deficient liquid media to select for the pRS314*dna2* mutant and to allow loss of the *URA3* containing the p*GAL*::*DNA2* plasmid. The resulting cultures were spotted on tryptophan-deficient or tryptophan-deficient media containing 5-FOA and grown at 23°C to determine the ability of the dna2 mutants to support growth. *dna2Δ pif1Δ mre11-H125N* containing the plasmids pRS314, pRS314*DNA2*, pRS314*dna2K1080E* grew, but strains containing the plasmids pRS314*dna2E675A* (nuclease minus) did not grow on 5-FOA-containing plates (Figure 7). In this Mre11-nuclease deficient background, there is also reduced viability of the transformant containing the pRS314*dna2K1080E*,*E675A* (nuclease minus, helicase minus). We conclude that the viability of the Dna2-nuclease defective *dna2Δ pif1Δ* (pRS314*dna2E675A*) at 23°C shown above (Figure 6) depends on a functional Mre11 nuclease, suggesting their complementarity not only during IR repair but also during DNA replication or repair of replication errors. This is consistent with a model in which Dna2 helicase makes a potentially toxic structure during DNA replication that is preferentially and coordinately processed by the Dna2 nuclease, but that can also be processed by Mre11 nuclease. Inactivation of Dna2 helicase is sufficient to remove the requirement for nucleolytic processing in the absence Dna2 alone but is insufficient in the absence of both Dna2 and Mre11 nuclease. In the *dna2Δ pif1Δ mre11-H12N* mutant, the residual MRX helicase may now contribute to generating a structure needing nuclease processing.

## Discussion

We have shown that the Dna2 5′ to 3′ nuclease can function in IR repair in the absence of an active Mre11 nuclease. The key result is that the *dna2Δ pif1Δ* strain is resistant to X-rays but the *dna2Δ pif1Δ mre11-H125N* mutant is as sensitive to X-rays as an *mre11Δ* strain, showing that in the absence of both the Mre11 nuclease and Dna2 nuclease, cells are nearly blocked in repair. Thus, strikingly, either nuclease can support repair efficiently (Figure 3). The most direct mechanistic inference is that Dna2 and Mre11 are performing at least partially overlapping nucleolytic functions in processing the ends of DSBs. Since Mre11 has 3′ to 5′ nuclease rather than 5′ to 3′ activity in vitro, the question arises as to how it can perform 5′ to 3′ nucleolytic degradation in the absence of Dna2. Mre11 may achieve this in the absence of Dna2 by cleaving hairpin structures in ends that are unwound by an unknown helicase [8]. Another interpretation is that Mre11 nuclease and Dna2 function sequentially and that a second nuclease, exonuclease 1, can substitute for Dna2 nuclease, when Mre11 is present. In the absence of Mre11 nuclease, however, Dna2 but not Exo1, can overcome the requirement for the Mre11 nucleolytic step. We favor the latter interpretation (see below).

While our studies show that the Dna2 nuclease can compensate entirely, in terms of repair and survival, for the absence of Mre11 nuclease, Dna2 cannot compensate for the absence of Mre11. Mre11 is clearly required to form the MRX end processing complex.

The interaction between Mre11 and Dna2 at DSB damage is paralleled by the fact that Dna2, like Mre11, is found at telomeres, the linear ends of chromosomes that resemble DSBs [32]. The Mre11 nuclease is required during the de novo synthesis of telomeres at a DSB with a telomere seed sequence, though an *mre11* nuclease deficient mutant does not have short telomeres [42]. Dna2 also appears to be required for extending a short telomere, and its nucleolytic function may be at play [32], [43], [44]. Interestingly, *dna2Δ pif1Δ* mutants have short telomeres compared to *pif1Δ* mutants [34].

Unexpectedly, our study has also supplied genetic evidence that supports biochemical studies suggesting that the Dna2 helicase and nuclease activities are coupled. *dna2Δ pif1Δ* expressing a Dna2-E675A (helicase plus, nuclease minus) protein have a reduced maximum permissive growth temperature and they are sensitive to IR. However, *dna2Δ pif1Δ* expressing a helicase minus, nuclease minus double Dna2 mutant protein has no growth or repair defect, i.e. inactivation of the helicase removes the requirement for the nuclease (Figure 6). This suppression occurs only if Mre11 nuclease is functional (Figure 7). Therefore, we propose that the Dna2 helicase creates a structure during DNA replication and DNA repair that is potentially toxic unless coordinately processed by the nuclease of either Dna2 or, in its absence, Mre11. Further, the observed lethality at all temperatures and in the absence of DNA damage in *mre11-nuclease minus dna2Δ pif1Δ* expressing nuclease defective Dna2 leads to the hypothesis that Mre11 nuclease may play a role in Okazaki fragment processing or in repair of lethal endogenous DNA damage. A role for Mre11 in OFP is consistent with previous results showing that *mre11-H125N rad27* mutants are inviable [18].

Also worth emphasizing is the fact that our findings suggest an additional role for Dna2, besides 5′ resection, in X-ray repair. *dna2Δ pif1Δ* strains are as resistant to IR as wildtype (this work), whereas *dna2Δ PIF1* overexpressing FEN1 to maintain viability is as X-ray sensitive as *rad52Δ* [31]. Therefore, during IR repair, Pif1 may contribute to formation of a structure that is lethal unless processed by Dna2, and this function is not complemented by *MRE11*, *EXO1*, or *SGS1*. Furthermore, a *dna2-2 PIF1* mutant (nuclease plus, helicase defective) is 100 times more sensitive to IR than *DNA2 PIF1* (at 75 krads) [31]. However, the *dna2-2* gene complements the X-ray sensitivity of the *dna2Δ pif1Δ mre11-H125N* strain (Figure 3B). These latter two results appear at odds but could be explained if Dna2-2 protein can perform resection but not some other function necessary for X-ray resistance in the presence of *PIF1*, either during resection or during strand exchange.

Mechanistic studies that were published after our work was completed show that *dna2* mutants as well as *sgs1* mutants exhibit significantly delayed long-range resection in a Rad51-independent single-strand annealing (SSA) repair reaction at an enzymatically-induced DSB [45], [46]. Based on these studies and the enzymatic activities of Dna2 and Sgs1, we propose that our results imply an interaction between Mre11 nuclease and Dna2/Sgs1 during resection in Rad51-dependent homologous recombination. At the same time, however, our new results also raise a conundrum. The reduced exonuclease resection in the *dna2Δ pif1m2* mutant observed in the SSA studies does not appear to cause a defect in subsequent Rad51-dependent repair [46], since we show that the *dna2Δ pif1Δ* strain is as resistant to IR as a wild-type strain after 70 krads IR (35 DSB or 2 per 17 chromosomes). Similarly, *sgs1Δ* strains, though they show reduced SSA, are as resistant to IR as wild-type strains, even after 75 krads. Thus, the mechanisms at work during the long-range resection in SSA may not be entirely interchangeable with the resection pathway leading to strand exchange. In this regard it is interesting that Exo1, another 5′ to 3′ nuclease, appears able to compensate for Dna2 or Sgs1 deficiency in SSA [45], [46], but does not appear to be able to do so in IR repair, since *dna2 mre11-H125N* and *sgs1 mre11-H125N* mutants are as sensitive to IR as *mre11Δ*, even though Exo1 is present at normal levels and since *mre11 nuclease-defective exo1* double mutants are not significantly more sensitive to IR than *mre11 nuclease deficient* mutants. Previous studies showed that only 100–300 bp of homology are required for Rad51-dependent gene conversion [47], [48], which differs from SSA in that gene conversion requires strand invasion of a homologous sister chromatid or homolog. We propose that the Dna2/Sgs1 pathway is the major pathway of resection in homologous recombination.

The observed IR sensitivity of the *sgs1 mre11-H125N* mutant we report here, combined with the defect in resection in *sgs1Δ* demonstrated by others, may help explain both some of our previous and our current results. We have shown that helicase defective *dna2-2* mutants are very defective in X-ray repair, but the double *sgs1Δ dna2-2* mutant is significantly more sensitive to IR than *dna2-2*, even though *sgs1Δ* itself is X-ray resistant [31]. Thus, Dna2 may compensate for the absence of Sgs1 helicase, but Sgs1 does not fully compensate for a defective Dna2 helicase. We have previously shown that BLM, a human counterpart of Sgs1 helicase, complements the MMS sensitivity of *dna2-2* mutants as well as the lethality of *dna2-1* mutants [39], and this suppression can now be attributed to suppression of defects in DSB processing. We expect that overexpression of BLM should also suppress the IR-sensitivity of *dna2-2* mutants. Genetic evidence suggests Pif1 helicase is an inhibitor of the Sgs1 helicase [49]. Another possible role for Dna2 may be to counteract Pif1 inhibition of Sgs1 during the 5′ to 3′ processing step.

What do our results tell us about the role of Dna2 in endogenous damage repair? We have previously identified the DNA replication fork pause at the replication fork barrier (RFB) in the ribosomal DNA as a specific site of endogenous DNA damage requiring the combined activities of Dna2 and Sgs1 for repair. In the presence of Fob1, DNA replication forks pause at the RFB and a DSB arises, the occurrence of which is elevated in *dna2-2* and *sgs1Δ* mutants [40], [50]. Interestingly, Pif1 is also required for normal levels of pausing at the RFB and subsequent chromosomal breakage [51]. These endogenous DSBs and the inability to repair them contribute to the synthetic sickness/lethality in the *dna2-2 sgs1Δ* strain, since the sickness/lethality is suppressed by the *fob1Δ* mutation [40], [50]. A similar situation occurs with Ctf4, a replication/cohesion protein, in which the *dna2-2 ctf4Δ* mutant is lethal, but the *dna2-2 ctf4Δ fob1Δ* is viable and slow growing. The *dna2-2 ctf4Δ fob1Δ* mutant is significantly more sensitive to IR than either *dna2-2 fob1Δ* or *ctf4 fob1Δ* mutants [33]. Presumably *dna2-2 ctf4Δ* inviability also results from failure to repair endogenous DSBs at the RFB. Thus, Dna2 plays a role in repairing endogenous breaks in collaboration with Sgs1 and Ctf4 and this is likely a reflection of the role of Dna2 and Sgs1 in resection. It is probably this role of Dna2 that accounts for the remarkable observation that increasing the gene dosage of Dna2 by one copy leads to partial genome stabilization and is absolutely required for viability of a *mec1-21* strain [52].

In conclusion, while our results and those of Zhu *et al.* [46] clarify the multiple events occurring early in repair at DSBs, especially the previously unappreciated centrality of Dna2, they also highlight several fundamental questions for the future: First, how much resection is sufficient for repair and what is the rate-limiting step in repair? Second, what is the mechanism of resection per se? How do Mre11 and Dna2, which may form a link between DNA replication and repair, coordinate repair of endogenous DSBs occurring due to replication fork failure?

## Materials and Methods

### Strains and Plasmids

The plasmids *pRS314DNA2*, pRS314*dna2K1080E*, pRS314*dna2E675A*, and pRS314*dna2K1080E*,*E675A* have the 6 kb EcoRI fragment containing Dna2 or Dna2 with the indicated mutations cloned into pRS314*CEN TRP* at the EcoR1 site. The construction of the Dna2 containing fragment and further construction of the mutants was described previously [25]. The plasmid pSEY18GALDNA2, referred to herein as pGAL::*DNA2*, was described previously [25], except that in this work the *DNA2* gene carried an additional FLAG tag, MDYKDDDK, at the N terminus of the protein.

The plasmids pRS414*MRE11*, pRS414*mre11-D56N*, and pRS*mre11-H125N* (gift of K. Lewis, Texas State University, San Marcos, TX) contain the *MRE11* genes with about 800 bp 5′ to the ATG and 136 bp 3′ to the TAG contained on a Kpn1 Not1fragment. The KpnI NotI fragments containing *mre11-D56N* and *mre11-H125N* were cloned into the KpnI NotI site of the integrating plasmid, pRS306. The pRS306*mre11-D56N* and pRS306*mre11-H125N* plasmids were cut with SphI and transformed into yeast.

All *MRE11* and *DNA2* alleles used in this study are expressed under the control of their native promoters.

Strains used are described in Table 1.

**Table 1:** Strains.

BY4741	*MATa his3Δ1 leu2Δ0 met15Δ0 ura3Δ0*
BY4742	*MATα his3Δ1 leu2Δ0 met15Δ0 ura3Δ0*
MB120-5A	*4742 MATα bar1Δ::kanMX trp1Δ*
MB121	*4741 MATa pif1Δ::HIS3 bar1Δ::kanMX*
MB122-17C	*4741 MATa mre11Δ::natR bar1Δ::kanMX trp1Δ*
MB123-1D	*4742 MATα mre11Δ::natR trp1Δ*
MB124-2D	*4741MATa mre11Δ::natR pif1Δ::HIS3 bar1Δ::kanMX trp1Δ*
MB126	*4742 MATα mre11Δ::natR mre11-D56N::URA3 trp1Δ*
MB127	*4741 MATa mre11Δ::natR mre11-H125N::URA3 trp1Δ*
MB128	*4741MATa mre11Δ::natR mre11-D56N::URA3 pif1Δ::HIS3 bar1Δ::kanMX*
MB129	*4741 MATa mre11Δ::natR mre11-H125N::URA3 pif1Δ::HIS3 bar1Δ::kanMX*
MB203	*4741 MATa dna2Δ::kanMX pif1Δ::HIS3 trp1Δ*
MB213	*4741 MATa dna2Δ::natR pif1Δ::HIS3 trp1Δ*
MB161B	*4741 MATa dna2Δ::kanMX pif1Δ::HIS3 trp1Δ bar1Δ::kanMX*
MB161B-56	*4741 MATa dna2Δ::kanMX pif1Δ::HIS3 trp1Δ bar1Δ::kanMX mre11-D56N*
MB161B-56	(pRS414*MRE11*)
MB161B-56	(pRS314*DNA2*)
MB161B-125	*4741 MATa dna2Δ::kanMX pif1Δ::HIS3 trp1Δ bar1Δ::kanMX mre11-H125N*
MB161B-125	(pRS414*MRE11*)
MB161B-125	(pRS314*DNA2*)
MB161B-125	(pRS314*dna2-K1080E*)
MB161B-125	(pRS314*dna2-E675A*)
MB161B-125	(pRS314*dna2-K1080E*,*K673A*)
MB161B-125	(pRS314*dna2-2*)
MB130	*4741 MATa dna2Δ::kanMX pif1Δ::HIS3 trp1Δ bar1Δ::kanMX mre11Δ::natR* (pRS414*MRE11*)
MB131	*4741 MATa dna2Δ::kanMX pif1Δ::HIS3 trp1Δ bar1Δ::kanMX mre11Δ::natR* (pRS414*mre11-D56N*)
MB132	*4741 MATa dna2Δ::kanMX pif1Δ::HIS3 trp1Δ bar1Δ::kanMX mre11Δ::natR* (pRS414*mre11-H125N*)
MB133	*4741 MATα dna2Δ::kanMX pif1Δ::HIS3 trp1Δ bar1Δ::kanMX mre11Δ::natR mre11-D56N::URA3*
MB134	*4741 MATα dna2Δ::kanMX pif1Δ::HIS3 trp1Δ bar1Δ::kanMX mre11Δ::natR mre11-H125N::URA3*
MB135	*4742 MATα sgs1Δ::kanMX sae2Δ::natR*
MB136	*4742 MATα sae2Δ::natR*
MB137	*4742 MATα sgs1Δ::kanMX*
MB138	*4741 MATα sgs1Δ::kanMX mre11Δ::natR mre11-H125N::URA3*

### X-ray Treatment

Cells were treated with X-rays as previously described using a Pantak MKII X-ray machine operated at 20 mA and 70 kev [31]. The dosimetry was determined using a Radca^λ^; 0.6 cubic centimeter ion chamber (model 10X5-0.6, serial no. 9352) connected to a Radiation Monitor Controller (model 9010; serial no. 90-2910).
